# Multivariate Covalent Organic Frameworks for High‐Performance Ammonia Nitrogen Separation: Structure‐Property‐Function Relationships

**DOI:** 10.1002/advs.202501173

**Published:** 2025-04-01

**Authors:** Yunhui Zhang, Jinglin Liu, Tao Wang, Kean Zhu, Yifan Gu, Zihao Wang, Meng Zhang, Zijian Xu, Zhenhua Chen, Haitao Li, Wei Jin

**Affiliations:** ^1^ College of Environmental Science and Engineering Tongji University Shanghai 200092 China; ^2^ State Key Laboratory of Pollution Control and Resource Reuse Shanghai 200092 China; ^3^ Shanghai Institute of Pollution Control and Ecological Security Shanghai 200092 China; ^4^ Key Laboratory of Urban Water Supply Water Saving and Water Environment Governance in the Yangtze River Delta of Ministry of Water Resources Shanghai 200092 China; ^5^ Shanghai Synchrotron Radiation Facility Shanghai Advanced Research Institute Chinese Academy of Sciences Shanghai 201204 China; ^6^ Key Laboratory of Yangtze River Water Environment Ministry of Education Tongji University Shanghai 200092 China

**Keywords:** adsorption, ammonia nitrogen, cation capture, covalent organic framework, structure‐property‐function relationship

## Abstract

Adsorption‐based separation of cationic pollutants, typically ammonia nitrogen (NH_4_^+^−N), from water holds great potential for environmental decontamination and resource recycling. However, NH_4_^+^ is more challenging to adsorb than other cations due to its stable structure and relatively large ionic radius. In this study, a “multivariate” synthetic strategy is applied to construct covalent channels through rational encoding sulfonic acid groups to enhance NH_4_^+^ adsorption and to investigate the structure‐property‐function relationships of sulfonated covalent organic frameworks (COFs). The optimal sulfonic acid group density is 50%, with an adsorption capacity of 17.09 mg g^−1^ and an equilibrium time of 5 min, far surpassing most adsorbents. The crystallinity of COFs significantly enhances both adsorption capacity and kinetics. Surface area and hydrophilicity primarily increaseadsorption capacity, with minimal influence on kinetics. In contrast, a large pore size correlates negatively with adsorption capacity but facilitates kinetics. N K‐edge near‐edge X‐ray absorption fine structure spectroscopy validates atomic‐level adsorption mechanisms of ion exchange between NH_4_^+^ and Na^+^ at the ‐SO_3_Na site and the formation of hydrogen bonds (N─H─N and N─H─O) between H of NH_4_^+^ and pyrrolic N as well as O of carbonyl on COFs. This study provides directions for designing ultrafast and high‐capacity adsorbents for cation capture.

## Graphical abstract

Theoretically, this study provides new insights into the atomic‐level adsorption mechanisms and structure‐property‐function relationships of sulfonated COFs for boosting ammonia nitrogen separation. Practically, the findings of this study can guide the design and optimization of COFs and other porous crystalline adsorbents to enhance the capture and separation of cations from water for resource recovery.

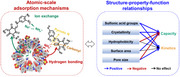

## 1 Introduction

The design of adsorbents for cation capture holds great significance and has far‐reaching implications in environmental protection, resource recovery,^[^ ^1^ ^]^ industrial processes,^[^ ^2^ ^]^ pharmaceutical production,^[^ ^3^ ^]^ and other fields. Typically, excessive ammonia nitrogen (NH_4_^+^−N), mainly originating from exogenous sources such as agricultural runoff, is a major contributor to water eutrophication and poses severe threats to aquatic ecosystems.^[^ ^4^ ^]^ However, ammonia nitrogen is also a valuable resource with diverse industrial applications, and its global production exceeds 200 million metric tons annually.^[^ ^5^ ^]^ Similarly, metal cations like lead, mercury, and even some rare earth elements^[^ ^6^ ^]^ in industrial wastewater are also dual‐natured as both pollutants and valuable resources. NH_4_^+^ was chosen as a model cation in this study due to its unique adsorption challenges in addition to its dual attributes. First, unlike commonly existing metal cations, which often possess complex electronic structures and readily form chemical bonds or electrostatic interactions with adsorbents, NH_4_^+^ has a tetrahedral structure with a relatively stable positive charge symmetrically distributed around the N atom.^[^ ^7^ ^]^ As a result, the adsorption of NH_4_^+^ is largely limited to weak electrostatic interactions and hydrogen bonding, while metal ions benefit from diverse adsorption mechanisms.^[^ ^8^ ^]^ Additionally, the relatively large ionic radius of NH_4_^+^ (0.154 nm)^[^ ^9^ ^]^ compared to metal ions such as Cu^2+^ (0.073 nm) and Hg^2+^ (0.102 nm)^[^ ^10^ ^]^ hinders its penetration into the pores of adsorbents and access to active sites. The adsorption of NH_4_^+^ is further complicated by its pH‐dependent conversion to NH_3_, necessitating strict pH conditions.^[^ ^7^, ^11^ ^]^ These challenges underline the complexity of designing adsorbents for NH_4_^+^ separation and highlight its utility as a model for advancing cation adsorption technologies.

The rational design and synthesis of high‐performance adsorbents are the keys to enhancing cation capture. Despite continuous and substantial efforts, the development of adsorbents that combine high adsorption capacity and fast kinetics remains a key challenge in this field.^[^ ^12^ ^]^ The adsorption performance of adsorbents is widely acknowledged to depend on their structure and physicochemical properties, such as surface area, pore characteristics, hydrophobicity, and binding sites.^[^ ^13^ ^]^ However, the intricate structure‐property‐function relationships of adsorbents are still inadequately understood. Moreover, the interaction among different properties can significantly affect adsorption efficacy. For example, introducing active sites like sulfonic acid groups enhances NH_4_^+^ adsorption but often compromises textural properties, such as surface area, due to steric hindrance, thus reducing overall performance.^[^ ^14^ ^]^ Therefore, balancing these trade‐offs is critical for designing adsorbents that excel in both adsorption capacity and kinetics. A deeper understanding of these interrelationships is essential to guide the rational design and optimization of adsorbents for NH_4_^+^ separation and beyond.

Covalent organic frameworks (COFs) have emerged as a promising class of crystalline porous materials,^[^ ^15^ ^]^ characterized by uniform nanoscale pores and modular building blocks,^[^ ^16^ ^]^ enabling precise functionalization, such as the introduction of sulphydryl,^[^ ^17^ ^]^ amidoxime,^[^ ^12^ ^]^ carbonyl,^[^ ^18^ ^]^ and ionic groups,^[^ ^12^ ^]^ to enhance adsorption performance.^[^ ^19^ ^]^ Parent and functionalized COFs have demonstrated exceptional efficiency in capturing metal cations^[^ ^20^ ^]^ and organic pollutants,^[^ ^21^ ^]^ far outperforming conventional adsorbents in terms of adsorption capacities, kinetics, and affinities. In addition, COFs have also been selected as a platform for elucidating the structure‐property‐function relationships in the adsorption of iodine,^[^ ^22^ ^]^ uranium,^[^ ^6^, ^12^ ^]^ and thorium.^[^ ^23^ ^]^ In this context, the design of stable COFs with diverse structures and tailored properties (hydrophilic channels and sulfonic acid groups with strongly acidic nature) offers a unique opportunity to elucidate the structure‐property‐function relationships for NH_4_⁺ adsorption.

Herein, we employed a multivariate design strategy, which involves the simultaneous manipulation of the density of functional groups, crystallinity, hydrophobicity, surface area, and pore characteristics, to construct covalent channels with rationally encoded sulfonic acid groups, thereby enhancing ammonia nitrogen separation and providing atomic‐level insights into adsorption mechanisms. A series of structurally diverse sulfonated COFs were designed and synthesized using a *p*‐toluenesulfonic acid (PTSA) salt‐mediated crystallization method^[^ ^24^ ^]^ to investigate the interplay of their properties and structure‐property‐function relationships. The proportion of sulfonic acid groups was tuned by introducing inert methyl groups, resulting in COFs designated as TpPaMePaSO_3_Na‐X (X = 0, 25, 50, 75, 100), with TpPaSO₃Na (X = 100) serving as a benchmark.^[^ ^25^ ^]^ Additionally, the effects of crystallinity, surface area, pore size, and hydrophobicity were systematically explored by varying reaction conditions, vacuum‐drying temperatures, and monomer structures. For example, TpPaSO₃Na≈150 was obtained by increasing the reaction temperature from 90 to 150 °C,^[^ ^24^ ^]^ while the specific surface area and pore size were tuned by adjusting vacuum‐drying temperatures (80 to 180 °C)^[^ ^26^ ^]^ and incorporating monomers with varying numbers of benzene rings, respectively. The hydrophobicity of the COFs was enhanced by methyl‐group substitution. Atomic‐level adsorption mechanisms were explored using N K‐edge near‐edge X‐ray absorption fine structure (NEXAFS) spectroscopy. This study is expected to guide the design and optimization of COFs and other porous crystalline adsorbents to enhance the capture and separation of cations such as NH_4_^+^ from aqueous environments.

## 2 Experimental Section

### 2.1 Materials

Benzidine (BD, >98%), 1,3,5‐triformylphloroglucinol (Tp, >97%), 2,5‐diaminobenzenesulfonic acid (Pa‐SO_3_H, 98%), 2,5‐diaminotoluene (PaMe, >98%), *p*‐phenyl‐enediamine (Pa, >97%), 4,4′‐diamino‐3,3′‐biphenyl disulfonic acid (BDSA, >97%), *p*‐toluenesulfonic acid (PTSA, >99%), acetone, absolute ethyl alcohol, and N,N‐dime‐thylformamide (DMF) were all purchased from Macklin company (Shanghai, China). The artificial ammonia nitrogen‐containing wastewater samples were prepared by dissolving ammonium chloride (NH_4_Cl, AR grade, Macklin, Shanghai, China) in deionized water. The solution pH was adjusted using HCl or NaOH solutions.

### 2.2 Preparation of COFs

**Figure 1:**
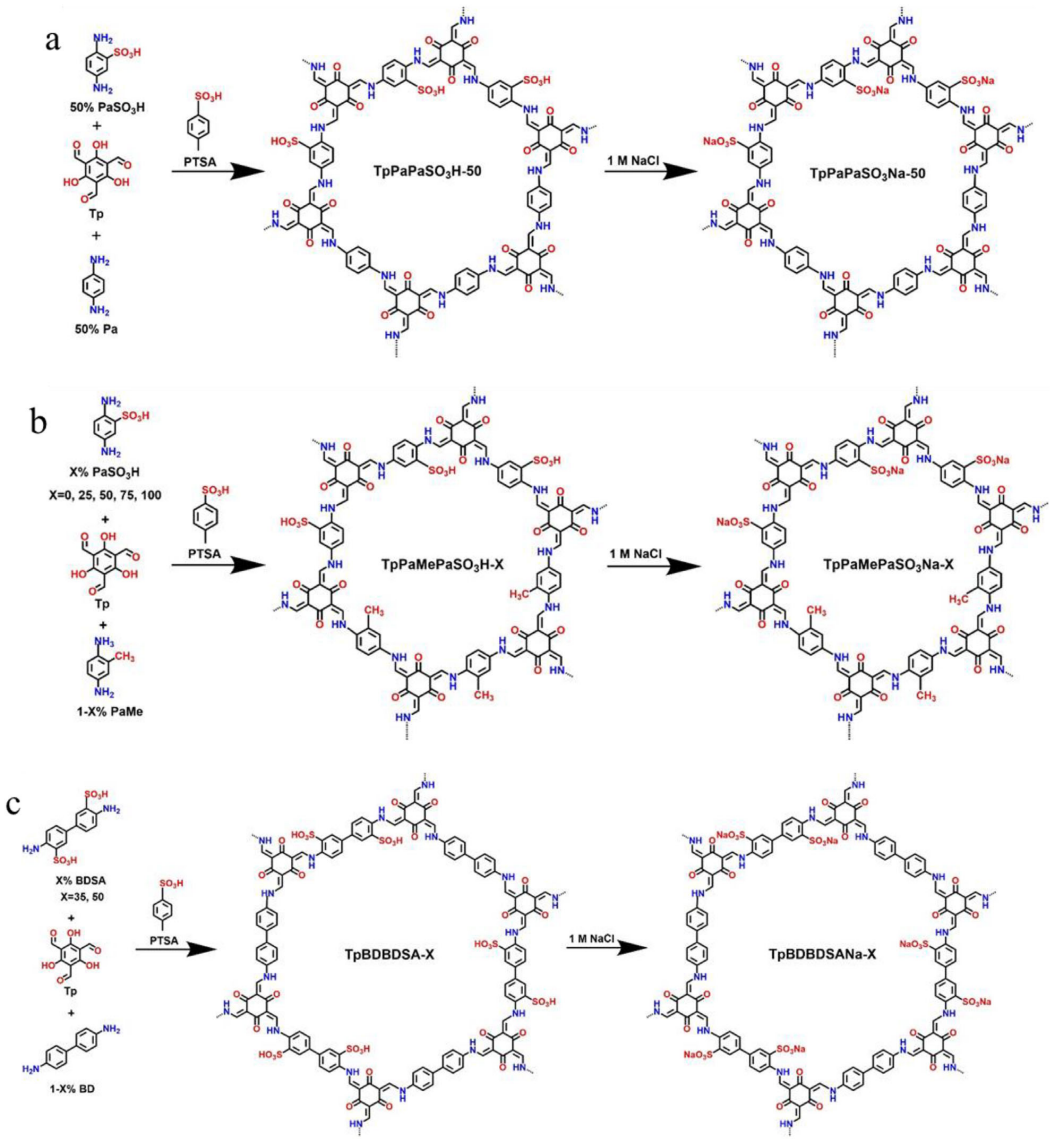
Schematic illustration of the synthesis of TpPaPaSO_3_Na‐50 a), TpPaMePaSO_3_Na‐X b), and TpBDBDSANa‐X c).

To elucidate the structure‐property‐function relationship of COFs for ammonia nitrogen adsorption, ten COFs with different structures and physicochemical properties were rationally designed and synthesized using a tridentate ligand synthesis strategy. Their detailed synthetic conditions are provided in Texts S1–S3 (Supporting Information). The PTSA catalytic grinding‐assisted method^[^ ^26^ ^]^ was adopted due to its distinct advantages (such as convenient operation and relatively low costs) for large‐scale production, facilitating practical applications. The synthesized COFs were classified into three groups based on ligands, namely TpPaPaSO_3_Na‐50, TpPaMePaSO_3_Na‐X, and TpBDBDSANa‐X, as shown in **Figure** **1** and Table S1 (Supporting Information).

### 2.3 Characterization

Powder X‐ray diffraction (XRD) analysis was conducted using a D/max‐Ultima IV diffractometer equipped with a Cu‐Kα ray source (λ = 1.54 Å). The scan parameters were set as follows: a scan size of 0.02°, a scan speed of 10° min^−1^, and a scan range from 3° to 30°. Fourier transform infrared (FT‐IR) spectra were collected using a Nicolet 6700 Thermo Smart FT‐IR spectrometer. The infrared spectral scan covered a range from 4000  to 400 cm^−1^, with a resolution of 0.09 cm^−1^ and 64 scans. N_2_ adsorption/desorption curves were obtained using a Mike ASAP3020 specific surface area analyzer. Prior to gas adsorption, all COF samples were degassed at 120 °C for 12 h. The specific surface area of COFs was determined by the Brunner Emmet‐Teller (BET) method, and the pore width distribution and pore volume were also obtained. The C, H, N and S contents in COFs were measured using an organic elemental analyzer (Elementar Unicube, Germany). N K‐edge near‐edge X‐ray absorption fine structure (NEXAFS) spectroscopy of TpPaPaSO_3_Na‐50 before and after ammonia nitrogen adsorption was obtained through soft X‐ray absorption spectroscopy (XAS) measurements at beamline BL08U1A of the Shanghai Synchrotron Radiation Facility. The element compositions on TpPaPaSO_3_Na‐50 before and after adsorption were analyzed by X‐ray photoelectron spectroscopy (XPS, Thermo Scientific K‐Alpha, USA) with a monochromatic Al Kα source (1486.6 eV). The in situ FTIR spectra of TpPaPaSO_3_Na‐50 during ammonia nitrogen adsorption (0, 1, 3, 5, 10, 20, and 30 min) was obtained by the FT‐IR spectrometer (Bruker INVENIO‐S, Germany). The contact angles of COF3 and COF7 were measured using a contact angle meter (JY‐82C, China).

### 2.4 Batch Adsorption Experiments

Adsorption performance was experimentally evaluated through adsorption kinetics and equilibrium adsorption capacity. The adsorption capacity of TpPaPaSO_3_Na‐50 was further quantified by adsorption isotherms. A total of 20 mg of the COF sample was added to 40 mL of an ammonium chloride solution with an initial concentration of 100 mgN L^−1^ (pH 7). The mixture was magnetically stirred at 400 rpm at room temperature. Approximately 3 mL of the suspension was sampled and filtered through a 0.22‐µm hydrophilic PTFE filter at time t = 0, 0.5, 1, 3, 5, 15, 30, 60, 120, and 1440 min for ammonia nitrogen measurement. The pseudo‐first‐order model and the pseudo‐second‐order model were applied to fit the adsorption kinetic data.^[^ ^27^ ^]^ To assess and compare the utilization of active sites for ammonia nitrogen adsorption, the utilization rate of active sites (i.e., sulfonic acid groups) was defined as η_SO3Na_ (see Text S2, Supporting Information) and calculated using Equation S1 (Supporting Information). The effects of solution pH were evaluated at initial pH values of 2, 3, 4, 5, 6, 7, 8, 9, and 10 under magnetic stirring at 400 rpm for 30 min. The adsorption selectivity was examined by cation competition tests using NaCl or CaCl_2_ solutions (0.05 and 0.5 mm, pH 7). The recyclability was evaluated by repeatedly eluting the ammonia nitrogen adsorbed on COFs with a NaCl solution, and cyclic adsorption tests were carried out for 5 cycles.

### 2.5 Statistical Analysis

The ammonia nitrogen concentration was measured using Nessler's reagent method with a UV–vis spectrophotometer (Hach DR6000, USA), and the detection limit was 0.025 mg L^−1^. For quality assessment and quality control, all adsorption tests were performed in duplicate at 20 °C, and blank tests were also conducted under identical conditions to account for ammonia nitrogen adsorption on filters and tubes. The crystallinity of COFs was calculated using Jade 6 software. Origin 2018 software was used to perform data fitting and modeling and to determine additional output parameters such as fitting values, standard errors, and correlation coefficients (R^2^).

## 3 Results and Discussion

### 3.1 High‐Performance Ammonia Nitrogen Separation by Sulfonated COF

**Figure 2:**
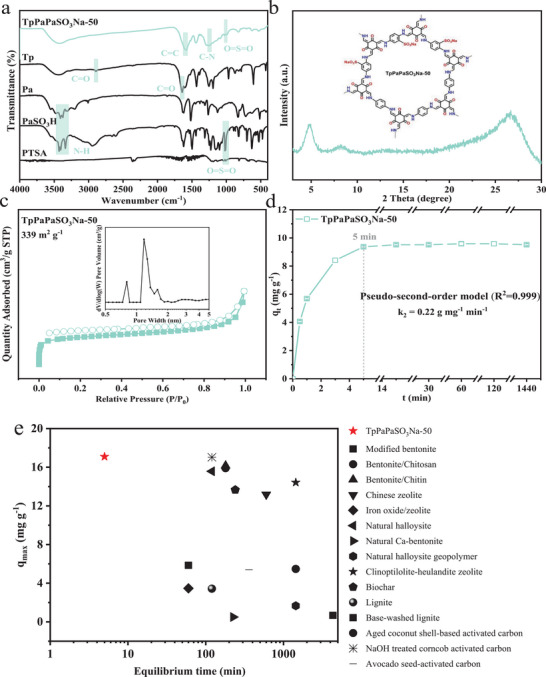
FT‐IR spectra a), XRD pattern b), N_2_ adsorption‐desorption isotherms and pore width distribution c) of TpPaPaSO_3_Na‐50, its adsorption kinetics of ammonia nitrogen d) and comparison with other adsorbents e).

TpPaPaSO_3_Na‐50 was successfully synthesized, featuring covalent channels and rational encoding of sulfonic acid groups (**Figure** **2**a–c). The FT‐IR spectrum (Figure 2a) shows the formation of the keto‐enamine linkage, evidenced by a C = C stretching absorption peak at 1577 cm^−1^ and a C‐N stretching absorption peak at 1258 cm^−1^.^[^ ^28^ ^]^ The porous crystalline structure of TpPaPaSO_3_Na‐50 was characterized by XRD and N_2_ adsorption‐desorption isotherms. The XRD pattern (Figure 2b) shows relatively intense peaks at 2θ = 4.9° and 26.4°, corresponding to the reflections of the (100) and (001) plane, respectively. The slightly broad peak at 26.4° might be attributed to defects in the π‐π stacking between consecutive COF layers.^[^ ^29^ ^]^ The N_2_ adsorption‐desorption isotherms at 77 K (Figure 2c) present typical type IV isotherms, indicating its porous structure. The average pore size was ≈1.3 nm, and the BET‐specific surface area was measured as high as 339 m^2^ g^−1^.

The adsorption kinetics of ammonia nitrogen on TpPaPaSO_3_Na‐50 are presented in Figure 2d. It was found that ultrafast adsorption kinetics were achieved within 5 min, with an equilibrium NH_4_^+^−N uptake capacity of 9.52 mg g^−1^. The maximum adsorption capacity was further determined to be 17.09 mg g^−1^ from the Langmuir model (R^2^ = 0.996) of the adsorption isotherms. The adsorption kinetics followed the pseudo‐second‐order model (R^2^ = 0.999), with a pseudo‐second‐order rate constant (k_2_) of 0.22 g mg^−1^ min^−1^, far surpassing most reported adsorbents under similar conditions (Figure 2e; Table S2, Supporting Information).^[^ ^25^ ^]^ The results of cyclic adsorption experiments (Figure S1a, Supporting Information) show no significant changes in ammonia nitrogen adsorption after 5 regeneration cycles, demonstrating high stability and recyclability, which are advantageous for its practical applications in water purification. Therefore, despite the complex processes and relatively high costs associated with the large‐scale synthesis of COFs compared to conventional adsorbents such as zeolites and biochars, TpPaPaSO_3_Na‐50 in this study still holds great potential for practical applications due to its extremely high adsorption capacity and kinetics, high stability, and recyclability, as well as the application of the PTSA catalytic grinding‐assisted method, which has distinct advantages (e.g., convenient operation and relatively low costs) in large‐scale production.

The ultrafast adsorption kinetics and high equilibrium adsorption capacity can be ascribed to the intrinsic structure and resulting physiochemical properties of COFs. For instance, the ordered COF channels can act as ion highways, sulfonic acid groups serve as readily accessible binding sites, and the high surface area exposes more active sites for ammonia nitrogen contact.^[^ ^30^ ^]^ To establish a paradigm for the development of ultrafast and high‐capacity adsorbents for ammonia nitrogen and other cations, the atomic‐level adsorption mechanisms and structure‐property‐function relationships were further investigated as follows.

### 3.2 Atomic‐Level Adsorption Mechanisms on Sulfonated COF

**Figure 3:**
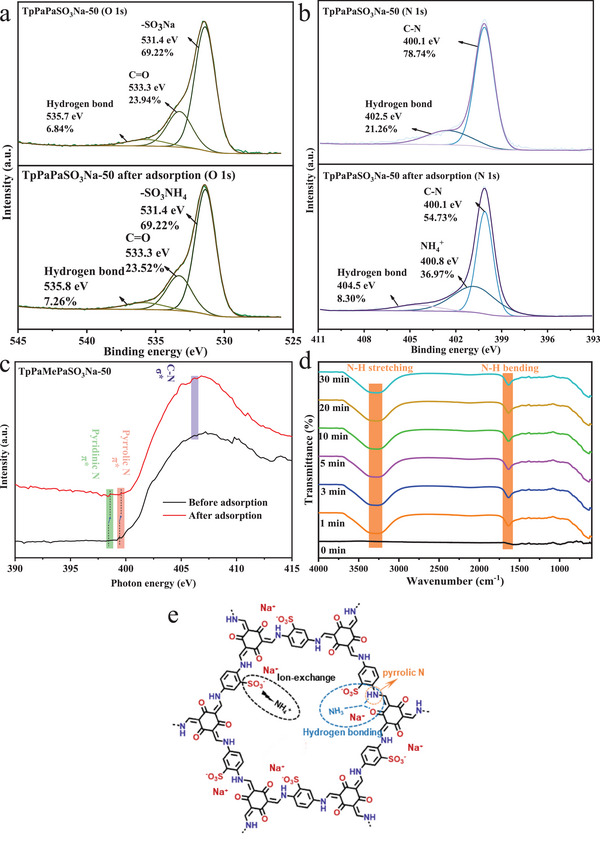
O 1s XPS spectrum a), N 1s XPS spectrum b), N K‐edge NEXAFS spectra c) and in situ FTIR spectra d) of TpPaPaSO_3_Na‐50 before and after ammonia nitrogen adsorption; Adsorption mechanism schematic diagram of ammonia nitrogen on TpPaPaSO_3_Na‐50 e).

To elucidate the atomic‐level adsorption mechanisms of NH_4_^+^ on sulfonated COFs, TpPaPaSO_3_Na‐50 was further characterized using XPS and NEXAFS analyses before and after adsorption. As shown in **Figure** **3**a, prior to adsorption, the O 1s XPS spectrum of TpPaPaSO_3_Na‐50 shows the presence of ‐SO_3_Na (531.4 eV), C = O (533.3 eV), and hydrogen bonds (535.7 eV), with atomic proportions of 69.22%, 23.94%, and 6.84%, respectively. After adsorption, the peak of ‐SO_3_NH_4_ (531.4 eV) emerged, with an atomic proportion of 69.22%, resulting from the cation exchange of NH_4_^+^ with Na^+^ at the ‐SO_3_Na active site on the sulfonated COFs. Additionally, the proportion of hydrogen bonds (535.8 eV) increased by 6.14%. The ion exchange between NH_4_^+^ and Na^+^ was also evidenced by the decrease in NH_4_^+^ adsorption with the increase in coexisting cations (Na^+^ and Ca^2+^) (Figure S1b, Supporting Information) and with the decrease in pH values due to competitive adsorption (Figure S1c, Supporting Information). The shift in hydrogen‐bond binding energy and the proportion of O atoms in hydrogen bonds were ascribed to the formation of hydrogen bonds between H atoms on NH_4_^+^ and O atoms on the COF.^[^ ^31^ ^]^ As shown in Figure 3b, before adsorption, the N 1s spectrum contained C‐N (400.1 eV) and hydrogen bonds (402.5 eV), with atomic proportions of 78.74% and 21.26%, respectively. After adsorption, it contained C‐N (400.1 eV), NH_4_^+^ (400.8 eV), and hydrogen bonds (404.5 eV), with atomic proportions of 54.73%, 36.97%, and 8.30%, respectively. The shift in the hydrogen‐bond binding energy was attributed to the formation of hydrogen bonds (N‐H‐N and N‐H‐O) between the H atom on NH_4_^+^ and the N atom of pyrrolic N as well as the O atom of the carbonyl on the COF. The appearance of the NH_4_^+^ characteristic peak confirmed the occurrence of ammonia nitrogen adsorption on the COF in the form of NH_4_^+^.^[^ ^32^ ^]^

NEXAFS analysis was carried out to further explore the changes in the electronic structure after ammonia nitrogen adsorption. As shown in Figure 3c, the N K‐edge spectra of TpPaPaSO_3_Na‐50 exhibited three resonance peaks, attributed to π* pyridinic N, π* pyrrolic N, and σ* C‐N, respectively.^[^ ^33^ ^]^ Interestingly, the typical peaks in N K‐edge spectra after adsorption showed a slight red shift compared to those before adsorption. The observed changes suggest a variation in the local environment around N and C atoms,^[^ ^34^ ^]^ which was attributed to the NH_4_^+^ adsorption and the formation of hydrogen bonds. In addition, the new presence of N‐H stretching (3280 cm^−1^) and N‐H bending (1630 cm^−1^) in the in situ FTIR spectra (Figure 3d) further confirmed the formation of new functional groups after ammonia nitrogen adsorption.^[^ ^35^ ^]^

Overall, the above results confirm that the adsorption process involved cation exchange between ammonia nitrogen (in the form of NH_4_^+^) and Na^+^ on the active site (‐SO_3_Na) of the COF and the formation of hydrogen bonds between H atoms on NH_4_^+^ and N atoms of pyrrolic N as well as O atoms of the carbonyl on the COF, as illustrated in Figure 3e.

### 3.3 Structure‐Property‐Function Relationships of Sulfonated COFs

#### 3.3.1 Density of Functional Groups

The effects of the density of functional groups on ammonia nitrogen adsorption by COFs were investigated through the introduction of sulfonic acid groups. This was achieved by controlling the ratio of two homologous monomers (i.e., Pa‐SO_3_H and Pa‐CH_3_) to obtain five sulfonated COFs (COF1 to COF5) with different densities of sulfonic acid groups. The density was indicated by the S content (wt.%), as presented in **Table** **1**. Theoretically, based on the molecular formula, the S content should be linearly related to the proportion of PaSO_3_H, denoted as X%. However, the measured S content was relatively lower than the theoretical value. This discrepancy was likely due to the non‐ideal reaction ratios of the two homologous monomers,^[^ ^14^, ^22^ ^]^ with MePa reacting more efficiently and having a higher utilization rate. The contents (both theoretical and measured values) of C, H, and N elements in the COFs are listed in Table S3 (Supporting Information).

**Table 1:** Structural parameters of TpPaMePaSO_3_Na‐X and their ammonia nitrogen adsorption performance.

No.	COF	S [wt.%]	BET [m^2^ g^−1^]	q_e_ [mg g^−1^]	η_SO3Na_ [%]
COF1	TpPaMePaSO_3_Na‐0	0	1064	0	NA
COF2	TpPaMePaSO_3_Na‐25	2.44	535	3.48	32.60
COF3	TpPaMePaSO_3_Na‐50	4.12	227	7.94	44.05
COF4	TpPaMePaSO_3_Na‐75	4.73	131	7.94	38.37
COF5	TpPaMePaSO_3_Na‐100 (TpPaSO_3_Na)	7.46	47	8.77	26.87

NA: not applicable.

**Figure 4:**
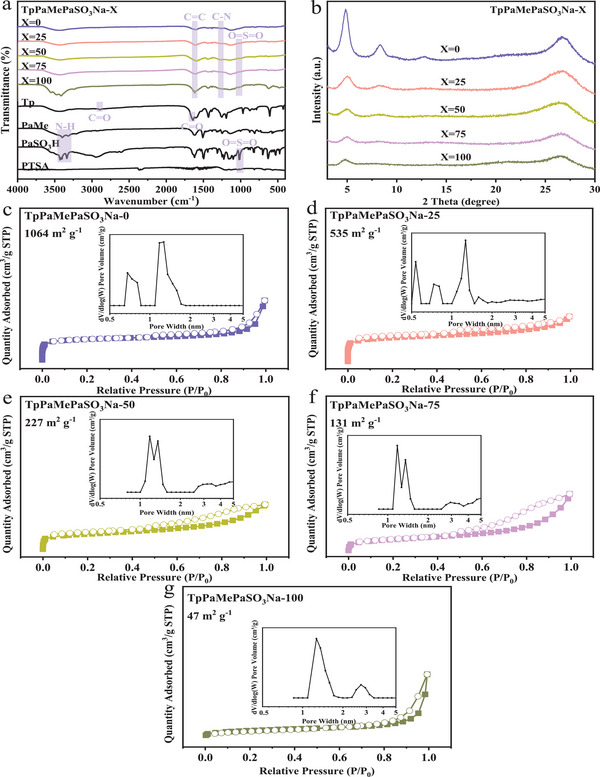
FT‐IR spectra a), XRD patterns b), N_2_ adsorption‐desorption isotherms, and pore width distribution c–g) of TpPaMePaSO_3_Na‐X samples (X = 0, 25, 50, 75, and 100).

The FT‐IR spectra in **Figure** **4**a confirm the reaction between Tp and diamine (MePa/Pa‐SO_3_H), namely the occurrence of the Schiff base reaction. The characteristic peaks of C = O (at 2891  and 1644 cm^−1^, respectively) and N‐H (at 3425  and 3333 cm^−1^, respectively) disappeared, while the characteristic peaks of C = C and C‐N (at 1600  and 1249 cm^−1^, respectively) appeared, demonstrating the formation of β‐ketoenamine‐linked framework structures.^[^ ^29^ ^]^ In addition, the absence of the peaks of sulfonic acid groups (at 1010 cm^−1^) in COF1 and the presence of stronger peaks with increasing X values in COFs indicate that the catalyst PTSA was thoroughly washed away and sulfonic acid groups were successfully incorporated into the COF chain.^[^ ^24^ ^]^ As shown in the XRD patterns (Figure 4b), the appearance of distinct peaks at ≈5° and 26°, corresponding to the 100 and 001 planes,^[^ ^29^ ^]^ respectively, indicates the highly ordered structure and high crystallinity of the synthesized COFs. The intensity of the characteristic peak at ≈5° gradually decreased as the sulfonic acid groups increased. This implies that the introduction of MePa was more conducive to high crystallinity, while the introduction of sulfonic acid groups reduced the crystallinity of COF.^[^ ^22b^ ^]^

All five COFs exhibited the typical type‐IV sorption isotherm at 77 K (Figure 4c), which is characteristic of mesoporous structure. Their specific surface area decreased from 1064 to 47 m^2^ g^−1^ as the S content increased from 0 to 7.46 wt.%, indicating that the introduction of sulfonic acid groups decreased the surface area of the COFs. The pore width of the COFs was concentrated at ≈1.27 nm, regardless of the proportions of sulfonic acid groups (Figure 4d). This is likely due to the fact that functional groups, including sulfonic acid groups and methyl groups, may mostly exist between COF layers rather than being restricted to hexatomic rings. Consequently, the quantitative change of bulky functional groups had little impact on the pore sizes of the COFs.^[^ ^28^ ^]^ The reduction in the crystallinity and surface area of the COFs after the incorporation of ionic sites is reasonable and has been frequently reported,^[^ ^14^, ^22^, ^28^ ^]^ probably due to the inhibitory effect of sulfonic acid groups on the crystallization of COFs.^[^ ^36^ ^]^

**Figure 5:**
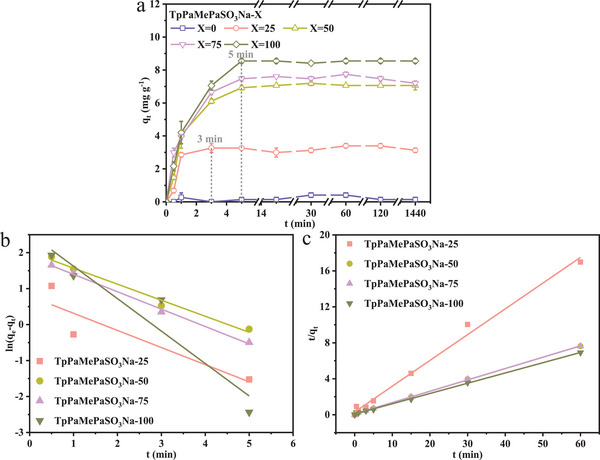
Adsorption kinetics of ammonia nitrogen on COFs a), linear fitting results by the pseudo‐first‐order model b) and pseudo‐second‐order model c).

The adsorption rate and equilibrium adsorption capacity were utilized to evaluate the adsorption performance of COFs. As shown in **Figure** **5**a, COF1 cannot adsorb ammonia nitrogen due to the absence of sulfonic acid groups and the chemical inertness of methyl groups. COF2 reached equilibrium within 3 min, slightly faster than other COFs with a higher proportion of sulfonic acid groups (which reached equilibrium within 5 min). The linear fitting of the kinetic data (Figure 5b,c; Table S4, Supporting Information) indicates that the pseudo‐second‐order model was the most suitable model, suggesting that the adsorption of ammonia nitrogen on TpPaMePaSO_3_Na‐X was chemisorption. The equilibrium adsorption capacity increased sharply from 3.48  to 7.94 mg g^−1^ as the proportion of sulfonic acid groups increased from 25% to 50%, and then increased steadily to 8.77 mg g^−1^ when the proportion reached 100%. In other words, when X>50, the increase in sulfonic acid groups had a negligible impact on the equilibrium adsorption capacity. To confirm the structure‐property‐function relationship of COFs, the efficiency of sulfonic acid group utilization by ammonia nitrogen, η_SO3Na_ (%), was defined as shown in Equation S1 (Supporting Information). As shown in Table 1, η_SO3Na_ reached a maximum of 44.05% when X was 50, significantly higher than 32.60% when X = 25, 38.37% when X = 75, and 26.87% when X = 100. This is reasonable because there is a trade‐off between the increase in active sites provided by the introduction of sulfonic acid groups and the consequent decrease in the crystallinity and surface area of COFs. Similar findings were also reported by Xie et al.^[^ ^22b^ ^]^ They constructed ionic covalent organic frameworks (iCOFs) for I_2_ adsorption, and the optimal material iCOF‐AB‐50 exhibited fast adsorption kinetics, good moisture tolerance, and full reusability, outperforming other iCOF‐AB‐X (X = 0, 33, 67, and 100, representing the proportion of *p*‐phenylene terephthalaldehyde monomer as active sites).

In summary, the introduction of sulfonic acid groups provides active sites for ammonia nitrogen adsorption within an appropriate ratio (such as ≈50% in this study). Simultaneously, the highly ordered channels of COFs can fully expose the sulfonic acid groups, enabling them to come into contact with and react with ammonia nitrogen. However, when the density of sulfonic acid groups is excessive, the crystallinity of COFs will deteriorate, and the specific surface area will decrease due to the occupation by sulfonic acid groups. This leads to a reduction in both the equilibrium adsorption capacity and the efficiency of sulfonic acid group utilization.

#### 3.3.2 Crystallinity

**Figure 6:**
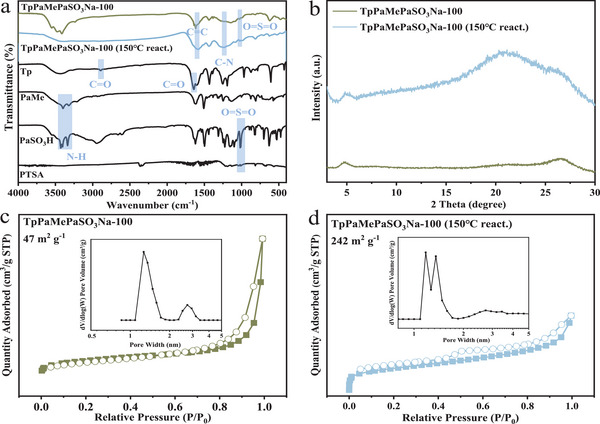
FT‐IR spectra a), XRD patterns b), N_2_ adsorption‐desorption isotherms and pore width distribution of TpPaMePaSO_3_Na‐100 (COF5) c) and TpPaMePaSO_3_Na‐100 (150 °C react.) (COF6) d).

Since the introduction of functional groups reduces the crystallinity of COFs, TpPaSO_3_Na (COF5) was chosen as a benchmark by removing the side group ‐CH_3_. TpPaSO_3_Na∼150 (COF6) was synthesized by raising the reaction temperature from 90 to 150 °C^[^ ^24^ ^]^ as a COF with poor crystallinity in comparison to COF5, aiming to investigate the relationship betwenn COF crystallinity and ammonia nitrogen adsorption. As shown in **Figure** **6**b, the peak at ≈5° in the XRD patterns nearly disappeared, confirming the loss of highly ordered structural features of COF6.

From the FT‐IR spectra in Figure 6a, the disappearance of the characteristic peaks of C = O (at 2891  and 1644 cm^−1^) and N‐H (at 3425  and 3333 cm^−1^), along with the appearance of C = C (at 1600 cm^−1^) and C‐N (at 1249 cm^−1^), reveals the formation of the β‐ketoenamine‐linked framework structures.^[^ ^29^ ^]^ This indicates the occurrence of the Schiff base reaction between Tp and the diamine monomer (Pa‐1 or Pa‐SO_3_H). The measured S content of COF6 (4.90 wt.%) was approximately half of that of COF5 (7.46 wt.%). This suggests that the increase in reaction temperature may have a negative impact on the introduction of sulfonic acid groups. The lower measured value compared to the theoretical one can be ascribed to the high activation energy of PaSO_3_Na, and the challenging process control, and non‐uniform surface distribution inherent in the catalytic grinding‐assisted synthetic method.^[^ ^24^, ^28^ ^]^ Both COFs exhibited typical type‐IV sorption isotherm profiles (Figure 6c) at 77 K, indicating a mesoporous structure. COF6, with lower crystallinity, had a higher surface area of 242 m^2^ g^−1^ compared to COF5 (47 m^2^ g^−1^). Their pore width distributions both concentrated at ≈1.27 nm (Figure 6d). In other words, a decrease in crystallinity may result in an increase in surface area and S content but has negligible effects on pore characteristics.

As shown in Figure S2 (Supporting Information), both COFs followed the pseudo‐second‐order model, which indicates chemisorption (R^2^ = 0.999). The equilibrium adsorption capacity of COF6 (4.95 mg g^−1^) was substaintially lower than that of COF5 (8.77 mg g^−1^), and the equilibrium adsorption time (t_e_) was extended three‐fold, from 5  to 15 min. The utilization efficiency of sulfonic acid groups also decreased from 26.87% to 23.09%, which was the lowest among the synthetic COFs in this study. This indicates that crystallinity is a fundamental factor for rapid adsorption and is one of the decisive factors for achieving high adsorption capacity. This can be explained by the fact that the highly ordered channels can facilitate the complete exposure of sulfonic acid groups and the rapid mass transfer of ammonia nitrogen from the external solution to the interior of COFs. This is consistent with previously reported findings that COFs exhibited a superior adsorption capacity and kinetics for iodine^[^ ^22b^ ^]^ and uranium^[^ ^6^, ^12^ ^]^ compared to an amorphous polymer (POP) with the same compositions. They attributed the fast kinetics of COFs to their layered pore structure and the uniform and dense distribution of chelate sites on pore walls, which facilitated the rapid diffusion of UO_2_^2+^ throughout the framework. Overall, the highly ordered structure of COFs is crucial for their fast adsorption, high adsorption capacity, high accessibility of sulfonic acid groups, and chemical stability. However, it should be noted that the maximum crystallinity of COFs is restricted by the catalytic grinding‐assisted synthetic method.

#### 3.3.3 Hydrophobicity

The isosteric substitution of hydroxyl groups (‐OH) with methyl groups (‐CH_3_, both molecular weight = 17 g mol^−1^) was applied as a balanced molecular design approach that preserves essential adsorption capacity while enhancing hydrophobicity. This is evidenced by comparative analysis of TpPaMePaSO_3_Na‐50 (COF3, contact angle = 37.77 ± 0.25°) and its hydroxyl‐containing counterpart TpPaPaSO_3_Na‐50 (COF7, contact angle = 31.40 ± 0.12°). It should be noted that although the methyl substitution induced a 20% hydrophobicity enhancement, the contact angle of COF3 remains below the conventional hydrophobicity threshold (>90°). This limitation is primarily attributed to the deliberately retained sulfonic acid groups (‐SO_3_H), which are essential for achieving the targeted ammonia nitrogen adsorption through ion‐exchange mechanisms. The adsorption kinetics show that the equilibrium adsorption capacity decreased from 12.24  to 10.21 mg g^−1^, while the equilibrium time remained unchanged as the hydrophobicity increased. This is reasonable because, in principle, hydrophobic pores impede the mobility and transport of polar cations such as ammonia nitrogen and their interactions with non‐polar pore surfaces.^[^ ^37^ ^]^ The hydrophobic microenvironment of the COF also disturbs proton‐transfer processes and further reduces hydrogen‐bonding interactions between NH_4_^+^ and polar functional groups on COFs.^[^ ^38^ ^]^ However, similar to the introduction of sulfonic acid groups, the introduction of methyl groups also inevitably decreased the crystallinity and surface area of COFs (from 339 to 227 m^2^ g^−1^), respectively. Therefore, aside from pore hydrophobicity, the decrease in adsorption performance may also be ascribed to the resulting decrease in the crystallinity and surface areas of COFs.

#### 3.3.4 Surface Area

**Figure 7:**
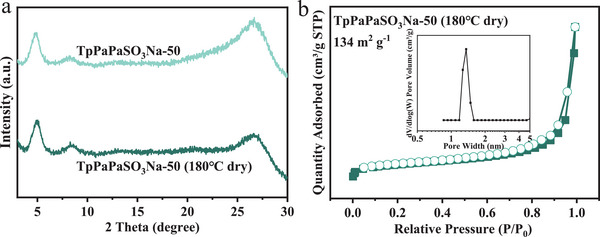
XRD patterns a), N_2_ adsorption‐desorption isotherms and pore width distribution b) of TpPaPaSO_3_Na‐50 (180 °C) (COF8).

The surface area of an adsorbent is a crucial parameter influencing its adsorption capacity.^[^ ^39^ ^]^ In this study, TpPaPaSO_3_Na‐50 (COF7) was used as a benchmark, and its surface area was reduced by varying the vacuum‐drying temperature^[^ ^26^ ^]^ from 80 to 180 °C to obtain TpPaPaSO_3_Na‐50∼180 (COF8) (339 m^2^ g^−1^ vs 134 m^2^ g^−1^) to investigate the effects of surface area. It was found that their S contents were similar, 3.91% for COF7 and 3.77% for COF8, respectively. However, the crystallinity increased, and their pore widths also increased from 1.18  to 1.48 nm after reducing the surface area (**Figure** **7**). In other words, it is nearly impossible to change only the surface area by increasing vacuum‐drying temperature because this inevitably increases the crystallinity and pore width. Therefore, the effects of surface area investigated here do not refer to its sole effects but also encompass the effects of the resulting altered properties, such as crystallinity and pore characteristics.

Similar to other COFs, the adsorption kinetics of COF7 and COF8 obeyed the pseudo‐second‐order model (R^2^ = 0.999), which indicates chemisorption (Figure S3, Supporting Information). Although both reached equilibrium at ≈5 min, the pseudo‐second‐order kinetic constant of COF8 was double that of COF7, being 0.5  and 0.22 g mg^−1^ min^−1^, respectively. This could be attributed to the increase in crystallinity, which is beneficial to adsorption kinetics. In terms of adsorption capacity, COF8, with a lower surface area, exhibited a lower equilibrium adsorption capacity (6.25 mg g^−1^ vs 9.92 mg g^−1^) and a lower sulfonic acid group utilization efficiency (37.89% vs 55.65%) despite having higher crystallinity. This suggests that an increase in the specific surface area is advantageous for enhancing the adsorption capacity and the utilization of active sites, as it increases the exposure of sulfonic acid groups and the accessibility of ammonia nitrogen, while having negligible effects on adsorption kinetics.

#### 3.3.5 Pore Size

**Figure 8:**
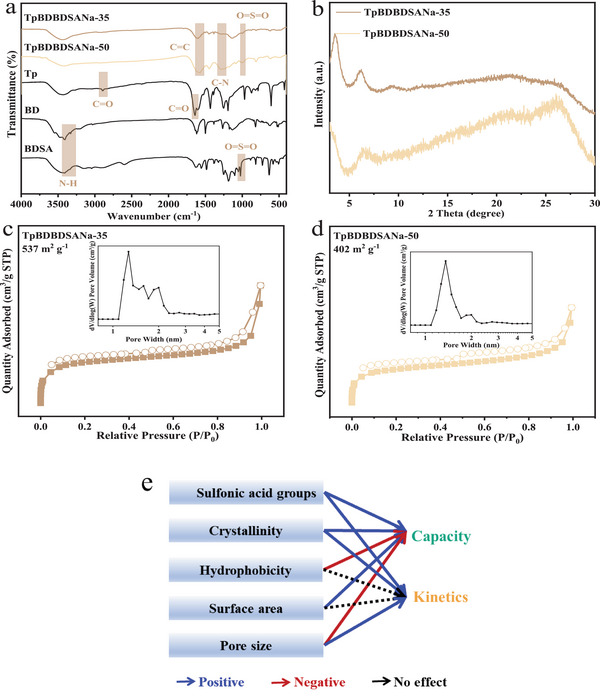
FT‐IR spectra a), XRD pattern b), N_2_ adsorption‐desorption isotherms and pore width distribution of TpBDBDSANa‐35 (COF9) c) and TpBDBDSANa‐50 (COF10) d), and summarized structure‐function relationship e).

To assess the effects of the pore size of COFs on ammonia nitrogen adsorption, the bi‐phenylenediamine monomer was used to replace the *p*‐phenylenediamine monomer in TpPaPaSO_3_Na‐50 (COF7) in different proportions, yielding TpBDBDSANa‐35 (COF9) and TpBDBDSANa‐50 (COF10) with pore sizes of 1.27  and 1.36 nm, respectively. The increase in S contents (1.96% and 4.10%, respectively) was consistent with the proportion of sulfonic acid groups (Table S3, Supporting Information), indicating the successful introduction of sulfonic acid groups, as corroborated by the FT‐IR spectra (**Figure** **8**). COF9 and COF10 both had mesoporous structures and high surface areas, 537 and 402 m^2^ g^−1^, respectively, compared to 339 m^2^ g^−1^ of COF7. However, it should be noted that diffraction peaks ≈ 3.6°, 6.1°, 9.4°, and 26.3° decreased and even disappeared in COF10, indicating its low crystallinity. In other words, the increase in the bi‐phenylenediamine monomer led to a severe destruction of the crystallinity of COFs compared to the *p*‐phenylenediamine monomer, which increased the synthesis difficulties and limited the practical applications of biphenyl‐sulfonated COFs. Overall, the increase in pore size increased the S content and surface area but significantly reduced the crystallinity of COFs.

The adsorption kinetic results (Figure S4, Supporting Information) show that the adsorption on COF9 and COF10 both reached equilibrium within 3 min, which was faster than the 5‐min equilibrium time for COF7. This was also evident from their higher k_2_ values, 2.31  and 0.63 g mg^−1^ min^−1^, respectively, compared to 0.22 g mg^−1^ min^−1^ for COF7. However, their equilibrium adsorption capacities were lower (5.52  and 6.94 mg g^−1^, respectively) than that of COF7 (9.52 mg g^−1^). The relatively higher adsorption capacity of COF10 might be ascribed to its higher S content. Nevertheless, its lower utilization rate of sulfonic acid groups (38.69% versus 64.37%) could be attributed to its extremely low crystallinity. Despite the relatively small difference in pore size between COF9 (1.27 nm) and COF10 (1.36 nm), which was restricted by the applied tridentate ligand synthesis strategy, the above results indicate that when the pore width of COFs was enlarged, the equilibrium adsorption capacity and sulfonic acid group utilization decreased, while the adsorption kinetics were enhanced. This is consistent with the findings of Sun et al.^[^ ^12^ ^]^ who introduced amidoxime as the chelating group into COF and expanded the pore width by increasing the number of benzene rings in the monomer for higher uranium removal. They explained the higher adsorption rate with larger open channels in terms of enhanced mass diffusion. Overall, the structure‐function relationship of NH_4_^+^ adsorption on sulfonated COFs is summarized in Figure 8e.

## 4 Conclusion

This study successfully constructed synthetic covalent channels through rational encoding sulfonic acid groups to enhance ammonia nitrogen adsorption and investigated the structure‐property‐function relationships of sulfonated COFs, aiming to guide the rational design of high‐performance adsorbents for cation capture. Although it is challenging to vary only one property of COFs to evaluate its sole effect on adsorption performance, some distinct structure‐property‐function relationships can be deduced. Sulfonic acid groups provide active sites for ammonia nitrogen but simultaneously reduce the crystallinity and surface area of COFs with their optimized loading at ≈50%, and therefore a balance among these factors is essential for attaining optimal adsorption capacity and kinetics. High crystallinity is beneficial for both the adsorption capacity and kinetics of NH_4_^+^. A high surface area and hydrophilicity are advantageous for adsorption capacity but have negligible impacts on kinetics, while pore size is negatively correlated to adsorption capacity but improves kinetics. The adsorption mechanisms were confirmed at the atom level as NH_4_^+^ cation exchange with Na^+^ at the ‐SO_3_Na active site on sulfonated COFs, as well as the formation of hydrogen bonds (N‐H‐N and N‐H‐O) between the H atoms of NH_4_^+^ and the N atoms of pyrrolic N as well as the O atoms of the carbonyl on sulfonated COFs. The findings of this study are expected to offer a new trade‐off paradigm for the development and functionalization of ultrafast and high‐capacity adsorbents for cation capture in the future, especially when considering the complex interactions among material structure, functional groups, and physiochemical properties for different cations.

## Conflict of Interest

The authors declare no conflict of interest.

## Supporting information



Supporting Information

## Data Availability

The data that support the findings of this study are available from the corresponding author upon reasonable request.
