# Supplementary material for: Multivariate Covalent Organic Frameworks for High‐Performance Ammonia Nitrogen Separation: Structure‐Property‐Function Relationships
Source: Adv Sci (Weinh). 2025 Apr 1;12(25):2501173. doi: 10.1002/advs.202501173 (PMC12224989; doi:10.1002/advs.202501173)
Supplement: Supplementary file 1 — Supporting Information [file ADVS-12-2501173-s001.docx]

### Supporting Information

### Multivariate Covalent Organic Frameworks for High-Performance Ammonia Nitrogen Separation: Structure-Property-Function Relationships

Yunhui Zhang 1,2,3,4, Jinglin Liu 1, Tao Wang 1, Kean Zhu 1, Yifan Gu 1,2, Zihao Wang 1, Meng Zhang 1, Zijian Xu 6, Zhenhua Chen 6, Haitao Li 6, Wei Jin 1,2,3,5*

^1^ College of Environmental Science and Engineering, Tongji University, Shanghai, 200092, China

^2^ State Key Laboratory of Pollution Control and Resource Reuse, Shanghai, 200092, China

^3^ Shanghai Institute of Pollution Control and Ecological Security, Shanghai, 200092, China

^4^ Key Laboratory of Urban Water Supply, Water Saving and Water Environment Governance in the Yangtze River Delta of Ministry of Water Resources, Shanghai, 200092, China

^5^ Key Laboratory of Yangtze River Water Environment, Ministry of Education, Tongji University, Shanghai, 200092, China

^6^ Shanghai Synchrotron Radiation Facility Shanghai Advanced Research Institute, Chinese Academy of Sciences, Shanghai 201204, China

**AUTHOR INFORMATION**

***Corresponding Author**

E-mail address: tjjinwei@tongji.edu.cn

Tel: +86 21 65976761

**Content**

**Text S1.** Synthesis of TpPaPaSO_3_Na-50

**Text S2.** Synthesis of TpPaMePaSO_3_Na-X

**Text S3.** Synthesis of TpBDBDSANa-X

**Text S4.** Calculation of adsorption parameters

**Figure S1.** Ammonia nitrogen adsorption on TpPaPaSO_3_Na-50 after consecutive regeneration cycles (a); Effects of coexisting cations (Na^+^ and Ca^2+^) (b) and solution pH (c)

**Figure S2.** Adsorption kinetics of ammonia nitrogen on TpPaMePaSO_3_Na-100 (COF5) and TpPaMePaSO_3_Na-100 (150℃ react.) (COF6)

**Figure S3.** Adsorption kinetics of ammonia nitrogen on TpPaPaSO_3_Na-50 (COF7) and TpPaPaSO_3_Na-50~180 (COF8)

**Figure S4.** Adsorption kinetics of ammonia nitrogen on TpBDBDSANa-35 (COF9) and TpBDBDSANa-50 (COF10)

**Table S1.** Summary of COF preparation conditions

**Table S2.** Adsorption performance comparison of TpPaPaSO_3_Na-50 with other adsorbents [2]

**Table S3.** Theoretical and measured values of C, H, N and S contents of COFs

**Table S4.** Linear fitting results of the pseudo-first-order model and pseudo-second-order model

**Text S1. Synthesis of TpPaPaSO_3_Na-50**

TpPaPaSO_3_Na-50 (COF7) was synthesized by replacing 2,5-diaminotoluene (MePa) with *p*-phenyl-enediamine (Pa) and X was set as 50. TpPaPaSO_3_Na-50-180 (COF8) was obtained by changing the vacuum-drying temperature from 80°C to 180°C.

**Text S2. Synthesis of TpPaMePaSO_3_Na-X**

Six TpPaMePaSO_3_Na-X samples (COF1 to COF6 in **Table S1**) were synthesized by adjusting the proportion of PaSO_3_H which is assigned as X% (X=0, 25, 50, 75 and 100). TpPaSO_3_Na was obtained when X=100 which has been proven as an ultrafast selective adsorbent for ammonia nitrogen [1]. The detailed synthesis protocol of COF1 to COF5 samples go as follows: Firstly, 2.5 mmol (475 mg) of *p*-toluenesulfonic acid (PTSA), (0.45*X%) mmol ((84.7*X%) mg) of 2,5-diaminobenzenesulfonic acid (Pa-SO_3_H) and (0.45*(1-X%)) mmol ((55.0*(1-X%)) mg) of 2,5-diaminotoluene (MePa) were transferred to an agate mortar (inner diameter=11 cm) and ground thoroughly for 5 min. Then, 0.3 mmol (63 mg) of 1,3,5-triformylphloroglucinol (Tp) was added to the mortar and ground for another 10 min, after which 0~50 μL of deionized water was added followed by continuous grinding until a doughy solid was obtained. After it was heated at 90°C for 24 h and ground into powder, 500 mL of deionized water and 60 mL of N,N-dimethylacetamide (DMF) were added in turn and filtered. It was then Soxhlet extracted for 48 h using acetone and vacuum dried at 80°C overnight. The solid was dispersed in 1 M NaCl solution and shaken at 200 rpm for 2 h. After filtration, the solid was thoroughly washed using a mixture of ethanol and deionized water (v/v=1/1, 500 mL) before dried at 90°C overnight. The bright red solid was finally obtained as TpPaMePaSO_3_Na-X with a yield of 85% to 90%. TpPaSO_3_Na~150 (COF6) was obtained by adjusting the reaction temperature from 90°C to 150°C.

**Text S3. Synthesis of TpBDBDSANa-X**

TpBDBDSANa-35 (COF9) and TpBDBDSANa-50 (COF10) were obtained by replacing 2,5-diaminotoluene (MePa) with benzidine (BD) and replacing Pa-SO_3_H with 4,4'-diamino-3,3'-biphenyl disulfonic acid (BDSA), and X was set at 35 and 50, respectively.

**Text S4. Calculation of adsorption parameters**

In order to assess and compare the utilization of active sites for ammonia nitrogen adsorption, the utilization rate of active sites (i.e., sulfonic acid groups) was defined as $\text{η}_{\text{SO}_{\text{3}}\text{Na}}$ (**Equation S1**).

$$\text{η}_{\text{SO}_{\text{3}}\text{Na}}\text{=}\frac{{\text{q}_{\text{e}}}/{\text{M}\text{w}_{\text{N}}}}{{\text{10ω}_{\text{S}}}/{\text{M}\text{w}_{\text{S}}}}\text{×100\% }\text{ }\text{ (S}\text{1}\text{)}$$

where $\text{η}_{\text{SO}_{\text{3}}\text{Na}}$ is the utilization of active sites (%); q_e_ is the adsorption amount of ammonia nitrogen by the adsorbent at adsorption equilibrium (mg g^-1^); ω_S_ is the mass fraction of S element obtained from element analysis (%); Mw_S_ and Mw_N_ are the relative atomic mass of S and N, respectively (Mw_S_=32 and Mw_N_=14).

$$\text{q}_{\text{t}}\text{=}\frac{\text{C}_{\text{0}}\text{-}\text{C}_{\text{t}}}{\text{Q}}\text{ (S2)}$$

where q_t_ is the adsorption amount of ammonia nitrogen by the adsorbent at moment t (mg g^-1^), C_0_ and C_t_ are ammonia nitrogen concentration and time 0 (10 mg L^-1^) and t (mg L^-1^), and Q is the solid/liquid ratio of adsorbent (0.5 g L^-1^ in this study).

$$K_{d}=\frac{C_{s}}{C_{a}} \text{ (S3)}$$

where K_d_ is the distribution coefficient; Cs and Ca are the equilibrium concentration of ammonia nitrogen on COFs and in aqueous solution (mg L^-1^), respectively.

**Figure S1. Ammonia nitrogen adsorption on TpPaPaSO_3_Na-50 after consecutive regeneration cycles (a); Effects of coexisting cations (Na^+^ and Ca^2+^) (b) and solution pH (c)**

**Figure S2.** **Adsorption kinetics of ammonia nitrogen on TpPaMePaSO_3_Na-100 (COF5) and TpPaMePaSO_3_Na-100 (150℃ react.) (COF6)**

**Figure S3. Adsorption kinetics of ammonia nitrogen on** **TpPaPaSO_3_Na-50 (COF7) and TpPaPaSO_3_Na-50~180 (COF8)**

**Figure S4. Adsorption kinetics of ammonia nitrogen on TpBDBDSANa-35 (COF9) and TpBDBDSANa-50 (COF10)**

**Table S1. Summary of COF preparation conditions**

| No. | COF | Reaction temp. (℃) | Vacuum-drying temp. (℃) | Yield (%) |
| --- | --- | --- | --- | --- |
| COF1 | TpPaMePaSO_3_Na-0 | 90 | 80 | 85~90 |
| COF2 | TpPaMePaSO_3_Na-25 | 90 | 80 | 85~90 |
| COF3 | TpPaMePaSO_3_Na-50 | 90 | 80 | 85~90 |
| COF4 | TpPaMePaSO_3_Na-75 | 90 | 80 | 85~90 |
| COF5 | TpPaMePaSO_3_Na-100 (TpPaSO_3_Na) | 90 | 80 | 85~90 |
| COF6 | TpPaSO_3_Na~150 | 150 | 80 | 70 |
| COF7 | TpPaPaSO_3_Na-50 | 90 | 80 | 91 |
| COF8 | TpPaPaSO_3_Na-50~180 | 90 | 180 | 90 |
| COF9 | TpBDBDSANa-35 | 90 | 80 | 65 |
| COF10 | TpBDBDSANa-50 | 90 | 80 | 31 |

**Table S2. Adsorption performance comparison of TpPaPaSO_3_Na-50 with other adsorbents [2]**

| Adsorbent | Contact time (min) | q_max_ (mg g^-1^) |
| --- | --- | --- |
| Modified bentonite | 60 | 5.85 |
| Bentonite/Chitosan | 180 | 15.90 |
| Bentonite/Chitin | 180 | 16.16 |
| Chinese zeolite | 600 | 13.18 |
| Iron oxide/Zeolite | 60 | 3.47 |
| Natural halloysite | 120 | 15.58 |
| Natural Ca-bentonite | 225 | 0.50 |
| Natural halloysite geopolymer | 1440 | 1.66 |
| Clinoptilolite-heulandite zeolite | 1440 | 14.42 |
| Biochar | 240 | 13.66 |
| Lignite | 120 | 3.43 |
| Base-washed lignite | 4320 | 0.67 |
| Aged coconut shell-based activated carbon | 1440 | 5.47 |
| NaOH treated corncob activated carbon | 120 | 17.03 |
| Avocado seed-activated carbon | 360 | 5.40 |
| TpPaPaSO_3_Na-50 | 5 | 17.09 |

**Table S3. Theoretical and measured contents of C, H, N and S in COFs**

| No. | COF | Measured content (%) | | | | Theoretical content (%) | | | |
| --- | --- | --- | --- | --- | --- | --- | --- | --- | --- |
|  |  | N | C | H | S | N | C | H | S |
| COF1 | TpPaMePaSO_3_Na-0 | 9.18 | 56.77 | 5.89 | 0.00 | 12.39 | 69.03 | 4.42 | 0.00 |
| COF2 | TpPaMePaSO_3_Na-25 | 9.08 | 52.84 | 5.59 | 2.44 | 11.29 | 61.69 | 3.73 | 3.23 |
| COF3 | TpPaMePaSO_3_Na-50 | 7.68 | 46.68 | 4.9 | 4.12 | 10.37 | 55.56 | 3.15 | 5.93 |
| COF4 | TpPaMePaSO_3_Na-75 | 7.80 | 47.33 | 5.08 | 4.73 | 9.59 | 50.34 | 2.65 | 8.22 |
| COF5 | TpPaMePaSO_3_Na-100 (TpPaSO_3_Na) | 6.52 | 36.85 | 4.01 | 7.46 | 8.92 | 45.86 | 2.23 | 10.19 |
| COF6 | TpPaMePaSO_3_Na-100~150 | 7.74 | 49.62 | 4.35 | 4.90 |  |  |  |  |
| COF7 | TpPaPaSO_3_Na-50 | 7.82 | 45.19 | 4.70 | 3.91 | 10.65 | 54.75 | 2.85 | 6.08 |
| COF8 | TpPaPaSO_3_Na-50~180 | 8.00 | 45.15 | 4.67 | 3.77 |  |  |  |  |
| COF9 | TpBDBDSANa-35 | 6.90 | 62.34 | 5.50 | 1.96 | 7.79 | 60.10 | 3.14 | 6.23 |
| COF10 | TpBDBDSANa-50 | 6.09 | 54.72 | 4.26 | 4.10 | 7.18 | 55.38 | 2.82 | 8.21 |

**Table S4. Linear fitting results of the pseudo-first-order model and pseudo-second-order model**

| COF | Pseudo-first-order model | | | | Pseudo-second-order model | | |
| --- | --- | --- | --- | --- | --- | --- | --- |
|  | k_1_  (min) | q_e_  (mg g^-1^) | R^2^ | k_2_  (g mg^-1^ min^-1^) | | q_e_  (mg g^-1^) | R^2^ |
| COF2 | 0.48 | 2.21 | 0.631 | 2.69 | | 3.48 | 0.991 |
| COF3 | 0.45 | 7.52 | 0.977 | 0.27 | | 7.94 | 1.000 |
| COF4 | 0.48 | 6.55 | 0.996 | 0.19 | | 7.94 | 1.000 |
| COF5 | 0.90 | 12.55 | 0.862 | 0.17 | | 8.77 | 0.999 |
| COF6 | 0.18 | 3.25 | 0.989 | 0.19 | | 4.95 | 0.999 |
| COF7 | 0.92 | 10.38 | 0.948 | 0.22 | | 9.52 | 0.999 |
| COF8 | 0.47 | 3.82 | 1.000 | 0.50 | | 6.25 | 0.999 |
| COF9 | 1.66 | 13.74 | 0.949 | 2.31 | | 5.52 | 0.997 |
| COF10 | 0.50 | 3.97 | 0.984 | 0.63 | | 6.94 | 1.000 |

## References

[1] W. Jin, J. Liu, N. Huang, Z. Wang, Y. Zhang, Y. Peng, C. Gong, Y. S. Ok, Z. Xu, *ACS EST Eng.* **2023**, *3* (10), 1511.

[2] B. Han, C. Butterly, W. Zhang, J. Z. He, D. L. Chen, *J. Clean. Prod.* **2021**, *283*, 124611.
